# Coronavirus disease 2019-associated nephropathy in an African American patient: a case report and review of the literature

**DOI:** 10.1186/s13256-023-03888-z

**Published:** 2023-04-07

**Authors:** Vijaypal S. Dhillon, Ahmad Alkashash, Karolina Viquez-Beita

**Affiliations:** 1 Internal Medicine, Indiana University School of Medicine Muncie, Muncie, USA; 2 Department of Pathology, Indiana University, Indianapolis, USA; 3 Hospitalist Department, IU Health Ball Memorial, Muncie, USA

**Keywords:** COVID, COVID-19, COVAN, Collapsing glomerulopathy, Critical care, ICU, Acute kidney injury, Acute renal failure

## Abstract

**Background:**

Acute kidney injury is now recognized as a common complication of coronavirus disease 2019, affecting up to 46% of patients, with acute tubular injury as the most common etiology. Recently, we have seen an increase in cases of collapsing glomerulonephritis in patients with coronavirus disease 2019, also known as coronavirus disease 2019-associated nephropathy. It has been noted to be seen with a higher incidence in African American patients who are carriers of the *APOL1* variant allele.

**Case presentation:**

A 47-year-old African American male with a past medical history of asthma presented to the emergency department with complaints of intermittent chest pain, shortness of breath, and worsening confusion. On admission, he was found to be hemodynamically stable, but labs were significant for elevated creatinine and blood urea nitrogen, signifying acute kidney injury. He was admitted and taken for emergent dialysis. During his hospitalization, he was found to be positive for coronavirus disease 2019. Renal biopsy was done, which showed collapsing glomerulopathy, and the patient continues to require outpatient dialysis after discharge.

**Conclusion:**

Collapsing glomerulonephritis has emerged as a complication in patients with coronavirus disease 2019. This condition should be particularly suspected in African American patients who present with acute kidney injury, nephrotic-range proteinuria, and who are positive for coronavirus disease 2019. Current treatment options are limited to supportive treatment and renal replacement therapy. More clinical cases and trials are needed to better understand and improve therapeutic outcomes in these patients.

## Background

Acute kidney injury (AKI) is now recognized as a common complication of the coronavirus-19 (COVID-19), affecting up to 46% [1] of patients with acute tubular injury as the most common injury. In recent years we have seen an increase in cases of collapsing glomerulonephritis, also known as COVID-19-associated nephropathy (COVAN), with a higher incidence in the African American population, specifically those carrying the *APOL1* allele [2, 3]. It is theorized that COVID-19 triggers an inflammatory cascade that may affect the *APOL1* variant gene, causing glomerular impairment. The disease may range from mild progressive kidney disease to acute renal failure. Currently, the mainstay of treatment involves supportive measures and renal replacement therapy. The authors present a case in which COVAN occurred in an African American male who continues to require long-term renal replacement therapy.

## Case presentation

A 47-year-old African American male with a past medical history of asthma presented to the emergency department with complaints of intermittent chest pain, shortness of breath, and worsening confusion. The patient was afebrile. In the emergency department, vitals showed an oxygen saturation of 96% on room air, a heart rate of 122 beats per minute, and a respiratory rate of 22 breaths per minute. On physical examination, the patient appeared ill and drowsy. While able to answer questions, he required repeated arousing as he was somnolent. Lungs were clear on auscultation, and no murmur was appreciated. The abdomen was soft and non-tender. He denied any history of alcohol, tobacco, or illicit drug abuse. There was no family history of confusion, dementia, or kidney disease. However, he reported exposure to COVID-19 in the past week.

While hemodynamically stable on admission, labs were remarkable for acute kidney injury with a serum creatinine of 31 mg/dL (normal 0.7–1.3 mg/dL), blood urea nitrogen (BUN) of 210 mg/dL (normal 6–24 mg/dL), potassium level of 7.6 mmol/L (normal 3.6–5.2 mmol/L), and a calcium level of 21.5 mg/dL (normal 8.6–10.3 mg/dL). Urinalysis showed significant proteinuria with levels equal to or greater than 500 mg/dL, white blood cells (WBC) of 21–50/hpf, and red blood cells of 6–10/hpf. Urine studies were done, which showed a urine protein level of 8700 mg/dL. Urine protein electrophoresis was interpreted as a glomerular pattern with no monoclonal bands seen, often seen in nephrotic and nephritic syndromes. A serology panel was sent, and antineutrophil cytoplasmic antibody (ANCA), antinuclear antibodies (ANA), human immunodeficiency virus (HIV), and hepatitis panel were negative. Electrocardiogram (ECG) showed normal sinus rhythm with peaked T waves.

**Fig. 1:**
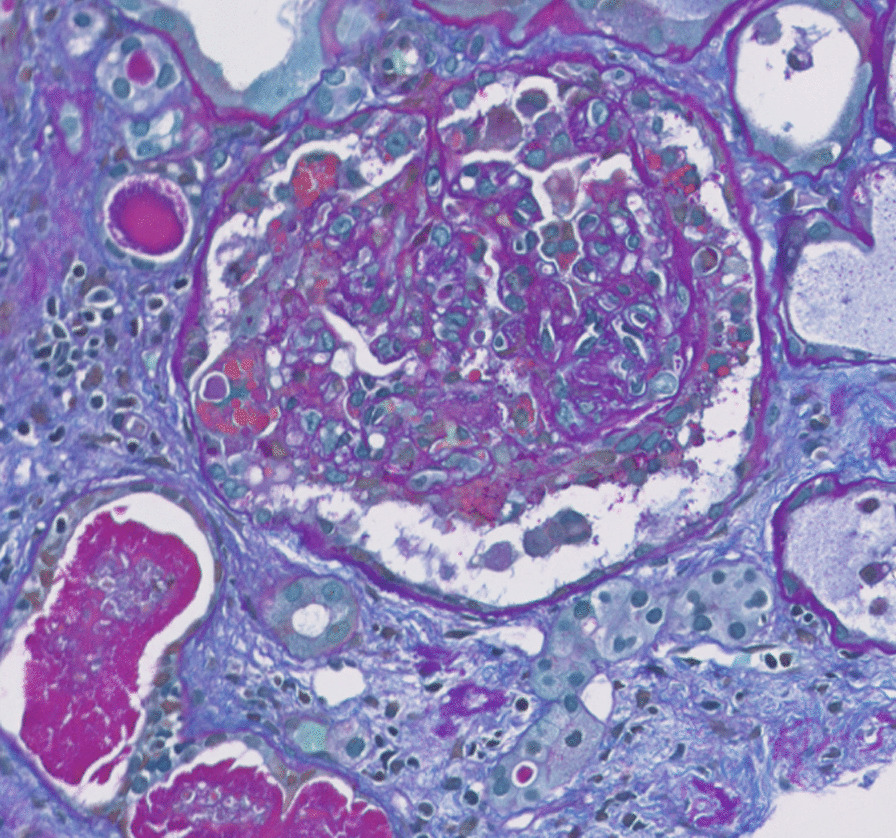
Allochrome: glomerulus with collapsing features; including collapse of basement membranes with segmental accentuation and overlying hyaline droplet filled epithelial cells. Active cellular crescents, however, are not seen. Mesangial matrix is mildly expanded. In addition, dilate tubules filled with hyaline and protein casts, loss of brush borders, and intraluminal cellular and granular debris are also seen

**Fig. 2:**
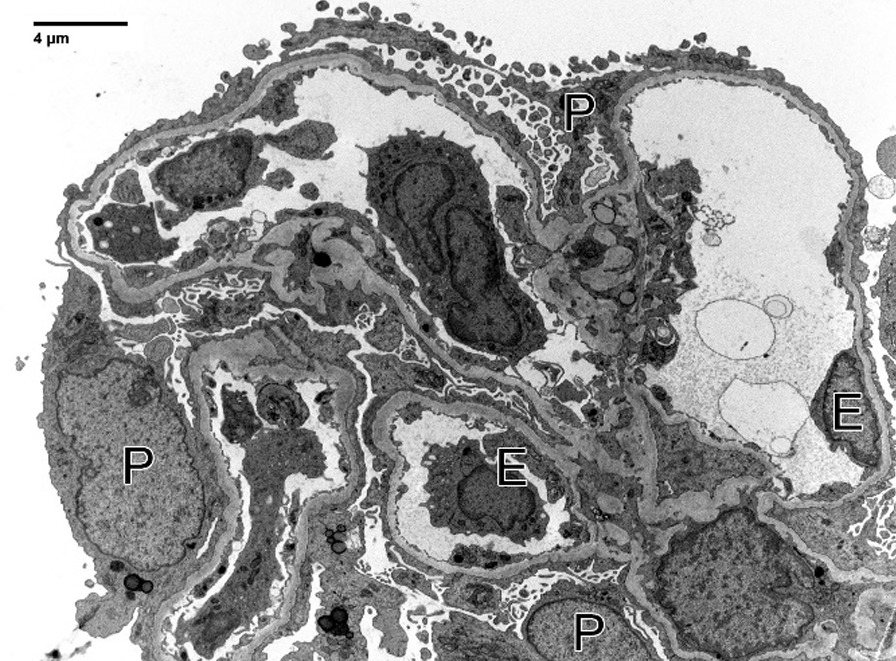
Electron microscopy: one non-obsolescent glomerulus was subjected to ultrastructural examination, with good tissue preservation. Podocytes display widespread effacement of foot processes, which correlates to proteinuria. Glomerular basement membranes are uniformly contoured except for a few segments with mild corrugation (mean glomerular basement membranes thickness, 303 ± 48 nm). Electron dense deposits were not seen

Further workup showed patient positive for COVID-19 by antigen test. As a result of the patient’s labs and condition, he was admitted and taken for emergent dialysis. A renal biopsy was performed, and light microscopy showed collapse of basement membranes in some glomeruli, along with severe tubular atrophy, interstitial fibrosis, and dilated tubules filled with hyaline and protein casts. Direct immunofluorescence did not show evidence of immune complex disease. Electron microscopy showed widespread effacement of foot processes, which correlated with proteinuria. These findings correlated with collapsing glomerulopathy (Figs. 1, 2). Computed tomography (CT) of the head and brain magnetic resonance imaging (MRI) scans were done, which did not show any acute findings. After dialysis, his encephalopathy showed significant improvement. He was more awake, more alert, and able to have extended conversations. Unfortunately, his kidney function did not improve, and he continues to require outpatient hemodialysis after discharge. Understandably, this has frustrated him, however, he has been compliant with follow-ups and dialysis appointments.

## Discussion

While cases of COVID-19 initially presented with severe respiratory distress syndrome, after vaccinations and the introduction of variants, the number of patients that are developing severe pulmonary disease has decreased, however, we have seen other complications such as renal impairment [1].

Focal segmental glomerulosclerosis is a major cause of end-stage renal disease. It can present with several variants, including collapsing, tip, cellular, perihilar, and not otherwise specified [4]. The collapsing variant of focal segmental glomerulosclerosis is described as segmental and global collapse of the glomerular capillaries, marked hypertrophy, and hyperplasia of the podocytes. It is a severe form with high risk of progression to irreversible kidney failure [5]. It was first described in patients with HIV infection [4], known as HIV-associated nephropathy (HIVAN). However, since the use and introduction of antiretroviral therapy, the incidence of HIVAN has decreased significantly. Collapsing glomerulopathy has also been associated with other viral infections, drugs, and ischemia [2, 5]. While other viral infections such as cytomegalovirus and Epstein–Barr virus have been associated with collapsing glomerulopathy, in recent years we have noticed an increased incidence of COVID-19-associated glomerulopathy, also known as COVAN. This pathology has been reported by several authors, and interestingly, all the affected patients are of African American descent with the *APOL1* high-risk genotype [2, 3, 5–15].

Currently, the pathology is not well understood. As high-risk patients with the *APOL1* genotype appear to be more affected, some authors believe COVAN parallels the development of HIVAN in patients with HIV. In HIVAN, it is believed that HIV causes a “second-hit” in patients with the *APOL1* genotype [2]. The infection, or inciting event, triggers an inflammatory response that activates the interferon–chemokine pathway. This in turn affects the *APOL1* variant gene, causing glomerular impairment. It is believed that COVID-19 triggers a similar inflammatory response and this is why patients with the *APOL1 *genotype are more prone to developing COVAN. The theory is supported by immunostaining of COVAN biopsies increasing the expression of phosphor-STAT3, which is activated by interleukin-6 [2, 7–9].

Another mechanism of injury that has been proposed is direct viral deposition in the glomerular membrane. This theory is controversial, given that viral particles are not present in some of the COVAN kidney biopsies [9].

Clinically, the patient presented with renal dysfunction and nephrotic range proteinuria with minimal respiratory symptoms. In several COVAN case reports, the outcome involves renal replacement therapy. The treatment for COVID-19 has been mainly directed toward acute respiratory failure, with medications such as dexamethasone, remdesivir, baricitnib, and toclizumab often indicated on the basis of oxygenation levels [16]. When patients present with COVAN but without hypoxemia, treatment is mainly supportive. Our case report and others raise the question of whether initiating these therapies in patients with COVAN will change the course of kidney failure and possibly prevent the need for long-term renal replacement therapies.

## Conclusions

COVAN has emerged as a complication in patients with COVID-19. This condition should be particularly suspected in patients of African American descent who present with COVID-19, acute kidney injury, and nephrotic-range proteinuria. *APOL1* variant carriers are at a higher risk of developing COVAN. More clinical trials are needed, especially in carriers of *APOL1*, to better understand the pathophysiology behind this disease and improve therapeutic options for these patients.

## Data Availability

The datasets during and/or analyzed during the current study are available from the corresponding author on reasonable request.
